# Auditory-motor entrainment and phonological skills: precise auditory timing hypothesis (PATH)

**DOI:** 10.3389/fnhum.2014.00949

**Published:** 2014-11-27

**Authors:** Adam Tierney, Nina Kraus

**Affiliations:** ^1^Auditory Neuroscience Laboratory, Northwestern UniversityEvanston, IL, USA; ^2^Institute for Neuroscience, Northwestern UniversityEvanston, IL, USA; ^3^Department of Communication Sciences, Northwestern UniversityEvanston, IL, USA; ^4^Department of Neurobiology and Physiology, Northwestern UniversityEvanston, IL, USA; ^5^Department of Otolaryngology, Northwestern UniversityEvanston, IL, USA

**Keywords:** synchronization, auditory timing, phonological skills, musical training, reading

## Abstract

Phonological skills are enhanced by music training, but the mechanisms enabling this cross-domain enhancement remain unknown. To explain this cross-domain transfer, we propose a *precise auditory timing hypothesis (PATH)* whereby entrainment practice is the core mechanism underlying enhanced phonological abilities in musicians. Both rhythmic synchronization and language skills such as consonant discrimination, detection of word and phrase boundaries, and conversational turn-taking rely on the perception of extremely fine-grained timing details in sound. Auditory-motor timing is an acoustic feature which meets all five of the pre-conditions necessary for cross-domain enhancement to occur (Patel, 2011, 2012, 2014). There is *overlap* between the neural networks that process timing in the context of both music and language. Entrainment to music demands more *precise* timing sensitivity than does language processing. Moreover, auditory-motor timing integration captures the *emotion* of the trainee, is *repeatedly* practiced, and demands focused *attention*. The PATH predicts that musical training emphasizing entrainment will be particularly effective in enhancing phonological skills.

## Musical training and reading

There is growing interest in the idea that music training can enhance language skills. This music-to-language transfer has been observed for a number of communication skills, including speech-in-noise perception (Parbery-Clark et al., 2009; Strait et al., 2012; Zendel and Alain, 2012) and verbal memory (Chan et al., 1998; Tierney et al., 2008; Parbery-Clark et al., 2009, 2011; Strait et al., 2010), but the skill that has been most extensively investigated is the ability to read. The hypothesis that musical training can enhance reading ability was tested as early as 1975 (Hurwitz et al., 1975) and has since been investigated in at least twenty-two separate studies. This work has ranged from cross-sectional comparisons of musicians and nonmusicians to longitudinal studies with random assignment of children to music training or control groups (for recent examples see Cogo-Moreira et al., 2013 and Rautenberg, 2013). Although there is a moderate degree of variability in the results reported, likely due to the heterogeneous nature of musical training, eighteen of the twenty-two studies reported that musical training increased reading or pre-reading abilities (for a review see Tierney and Kraus, 2013a).

Thus there is considerable evidence that musical training enhances reading skills. *Why* it can do so remains unknown. At first glance, musical training and the skills necessary for the acquisition of reading would appear to have little in common, as one requires auditory-motor integration, while the other would appear to be a silent visual activity. Complicating the search for an explanation of how these two domains of expertise are related is that both music performance and reading are complex skills which rely on a host of perceptual and cognitive processes. To develop a hypothesis for the mechanisms underlying transfer of learning from music to reading, therefore, one must first identify these musical and linguistic processes. Second, one must demonstrate that certain shared neural resources provide the foundation for both music and reading processes. If successful, such a hypothesis could enable the development of musical training regimens targeted to maximize transfer of learning to language skills.

## Auditory-motor entrainment and reading

Here we demonstrate the feasibility of this approach by focusing on entrainment to auditory signals. In this paper we use the term “entrainment” to refer to the process of moving to a repeated auditory signal such that there is a consistent relationship between the timing of one’s movements and the timing of sound onsets. Deficits in auditory-motor entrainment have been found alongside preserved discrimination of complex rhythms (Fries and Swihart, 1990), suggesting that auditory-motor entrainment is a skill dissociable from other rhythmic abilities (see the later section entitled “Other rhythmic skills”). Entrainment is also an integral part of group musical performance, and is therefore a skill which could be improved by a wide variety of musical training approaches. We argue that learning to entrain to complex stimuli may enhance the development of phonological awareness, the explicit knowledge of the component sounds of speech. Phonological awareness is a vital component of normal reading development and is impaired in poor readers (Ramus, 2003; Ramus et al., 2003; Rvachew and Grawburg, 2006; Siegel, 2006).

**Figure 1:**
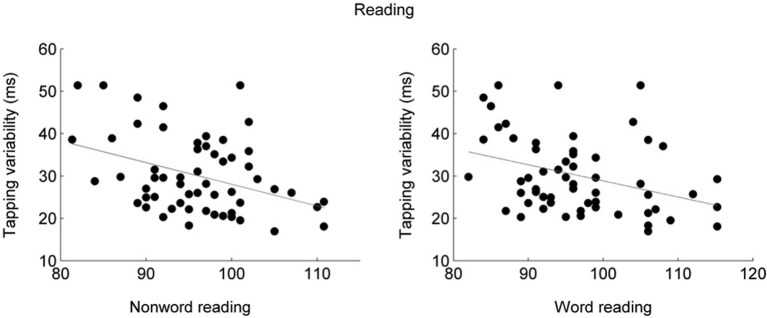
**Participants who tapped less variably to a metronome also performed better on tests of nonword reading (left; *r* = −0.35, *p* = 0.0067) and word reading (right; *r* = −0.38, *p* = 0.0036) tasks (Tierney and Kraus, 2013b)**. Synchronized tapping performance was measured as the average standard deviation across two speeds, 1.5 and 2 Hz. Word and nonword reading abilities were assessed using the Test of Word Reading Efficiency (TOWRE; Torgeson et al., 1999).

Participants who have difficulty reading have difficulty entraining to a metronome (Thomson et al., 2006; Thomson and Goswami, 2008; Goswami, 2011). This relationship between entrainment and reading skill has also been demonstrated in typically-developing participants (Tierney and Kraus, 2013b; see Figure 1). This relationship, therefore, is not specific to learning impairment and instead applies to reading skills more generally. Moreover, even before learning to read, children who are able to entrain to a metronome also have better language skills such as phonological awareness and verbal memory (Woodruff Carr et al., 2014), suggesting that entrainment is linked to core abilities necessary for the acquisition of reading. Entrainment skill is connected more strongly to language skills than the ability to tap steadily in silence (Thomson et al., 2006; Thomson and Goswami, 2008; Corriveau and Goswami, 2009; Tierney and Kraus, 2013b), suggesting that it is the ability to use auditory input to maintain and correct one’s timing that is important for reading acquisition. Aside from its relationship to language, entrainment variability is lower in trained musicians (Repp and Doggett, 2007; Repp, 2010), suggesting that it is a trainable skill.

## The OPERA hypothesis

Here we explore the question of why auditory-motor entrainment practice might lead to enhanced phonological skills and subsequent reading ability through the framework of the OPERA hypothesis (Patel, 2011, 2012, 2014). OPERA proposes that effects of musical training on language skills can be understood by examining shared acoustic features of music and speech and demonstrating that they satisfy five conditions: overlap, precision, emotion, repetition, and attention. First, the neural resources responsible for processing that acoustic feature in both music and speech must overlap, enabling transfer from learning in one domain to the other. Second, the precision with which the feature must be processed must be greater in music than in language, with the result that music training improves precision of processing in language. Third, the feature must elicit emotion when encountered in music, as emotion augments perceptual learning. Fourth, the feature must be repeatedly processed in the course of listening to or playing music, enabling the repeated practice that is crucial for learning. Finally, the feature must be the subject of directed attention.

We propose the Precise Auditory Timing Hypothesis (PATH): both auditory-motor entrainment and phonological awareness rely upon precise neural timing in the auditory system and integration of this auditory timing information with motor and cognitive networks. In the remainder of this paper we examine the five conditions of the OPERA hypothesis and demonstrate that *precise auditory timing* satisfies all five. We do not propose that precise auditory timing is the *only* shared feature driving the effects of musical training on reading ability. Given the heterogeneous natures of both music and reading, there are likely several mechanisms by which musical training can enhance reading. Nevertheless, our view is that PATH provides a framework to understand a principal mechanism of music-reading transfer.

## Overlap

### Perception of the timing of sound events, which is important for both acquiring phonological skills and entraining to music, relies on the precision of the auditory system’s processing of sound

When a participant moves at a consistent tempo in the absence of any perceptual input, their performance can be modeled as a simple combination of variability contributed by the motor system and variability contributed by an internal timekeeper (Wing and Kristofferson, 1973). When a pacing stimulus is present, however, a third component is added to the model—that of auditory-motor feedback. Small variations in motor timing, if not corrected, will tend to cause movement to drift away from the stimulus. In order to entrain, therefore, participants must perceive the timing of each sound, compare this information with the timing of their movement, and adjust their next motor movement accordingly (Semjen et al., 1998). Less accurate temporal perception may lead to a greater build-up of timing discrepancies prior to successful error correction, resulting in more variable performance (Krause et al., 2010). Participants who move less variably when entraining to a metronome are better able to distinguish two nearly simultaneous events (Krause et al., 2010), confirming that expert entrainment relies upon precise auditory timing perception.

The development of phonological awareness relies upon the ability to align precise perception of timing with meaningful phonological categories. For example, in consonant-vowel syllables, the phonemes /d/ and /t/ can be distinguished by their voice onset time (VOT), or the difference in time between the release of the stop (the movement of the tongue away from the roof of the mouth) and the onset of vowel voicing. To learn to perceive these phonemes listeners must track sound timing and, through experience, learn to classify sounds with a certain range of timing as belonging to one or the other phonological category. This learned classification then enables children to map letter combinations onto speech sounds in the course of learning to read. The development of phonological awareness, therefore, requires children to integrate auditory timing information with the cognitive and motor networks responsible for developing the ability to represent and produce speech sounds. Children with reading impairment have difficulty with this timing integration, as they often exhibit abnormal categorical perception of stop consonants (Reed, 1989; Kraus et al., 1996; King et al., 2002; Richardson et al., 2003; Serniclaes et al., 2004; Tsao et al., 2004; Sharma et al., 2006; Boets et al., 2008, 2011; Vandermosten et al., 2011; Berent et al., 2012).

Moreover, the perception of certain temporal patterns, such as the slowing that tends to occur as speakers approach the ends of sentences (Klatt and Cooper, 1975; Fant et al., 1991; Vaissière, 1991; Venditti and van Santen, 1998), can facilitate the perception of word and phrase boundaries (Nakatani and Schaffer, 1978; Smith et al., 1989; Cutler and Butterfield, 1992). Further confirming the importance of precise time perception for reading, children with language impairments also have problems with backward masking, the ability to perceptually separate a sound and a subsequent noise burst (Wright et al., 1997; Marler et al., 2001, 2002; McArthur and Hogben, 2001; Griffiths et al., 2003; Montgomery et al., 2005; Gibson et al., 2006; Tierney and Kraus, 2013b).

**Figure 2:**
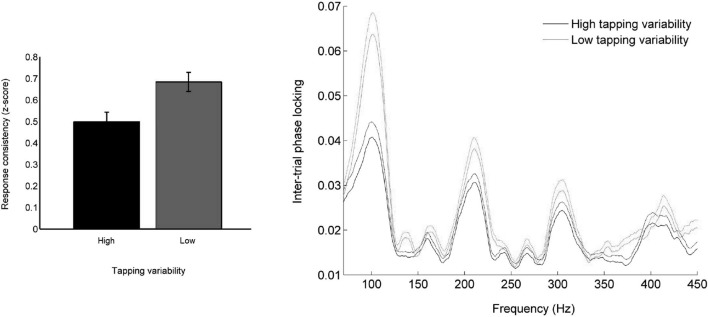
**(Left)** Participants who moved consistently to a metronome also tended to have consistent neural responses to speech. **(Right)** Less variable movement during entrainment is associated with neural responses to sound that are jittered less in time from trial to trial. Temporal jitter was measured by calculating frequency-by-frequency phase consistency across all trials (Tierney and Kraus, 2013c).

The inferior colliculus represents auditory timing with high temporal precision (Warrier et al., 2011) and connects directly to the cerebellum (Mower et al., 1979; Hashikawa, 1983; Saint Marie, 1996), a structure involved in auditory-motor entrainment (Rao et al., 1997; Molinari et al., 2007; Bijsterbosch et al., 2011; Grahn et al., 2011) and tracking of temporal structure in speech (Kotz and Schwartze, 2010; Schwartze and Kotz, 2013). This colliculo-cerebellar connection may, therefore, be a primary pathway by which auditory timing information is used as feedback to correct errors in motor output during entrainment. Participants who move more variably to a metronome also show greater trial-by-trial timing variability (Tierney and Kraus, 2013c; see Figure 2) and delayed onset timing (Tierney et al., 2014) of the brainstem response to sound (primarily generated within the inferior colliculus), suggesting that the precision with which timing information is represented in the subcortical auditory system helps determine the precision with which listeners can perceive, react to, and predict the timing of auditory events (see also Woodruff Carr et al., 2014). If an individual’s auditory system cannot generate responses to sound that occur with consistent, reliable timing, estimations of the timing of sound events that rely on these responses may be blurred, and this lack of precise temporal perception may affect the stability of entrainment to auditory signals.

**Figure 3:**
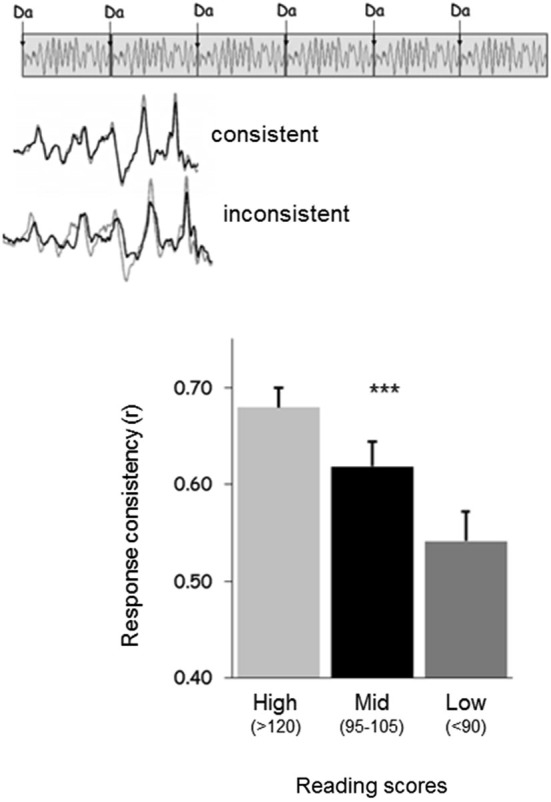
**(Top)** Response consistency is a neural measure that assesses the extent to which the neural response to sound is consistent on a trial-by-trial basis across a recording. **(Bottom)** Good readers have responses with greater consistency than average readers, who have responses with greater consistency than poor readers (Hornickel and Kraus, 2013).

**Figure 4:**
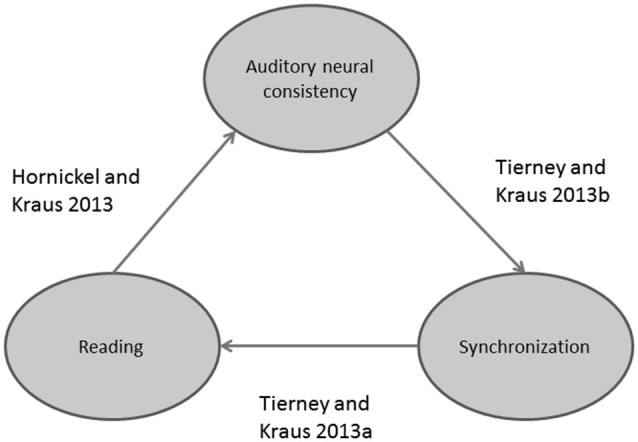
**Schematic displaying recently discovered relationships between reading, auditory-motor entrainment, and auditory neural consistency**.

Poor readers (i.e., subjects with poor phonological skills) also exhibit brainstem responses that are delayed (King et al., 2002; Banai et al., 2005, 2009) and more variable on a trial-by-trial basis (Hornickel and Kraus, 2013; see Figure 3) compared to those of good readers. Delayed and variable neural responses are likewise present in a rat model of dyslexia (Centanni et al., 2013). Thus, the exact timing perception necessary for both auditory-motor entrainment and phonological processing may depend on the temporal precision of the auditory system (Figure 4). As a result, repeated practice entraining to music could engender benefits for perception of the timing of speech sounds, eventually leading to enhanced phonological awareness.

## Precision

### Although both phonological skills and auditory-motor entrainment rely on precise timing, entrainment places greater demands on timing precision

The development of phonological awareness depends on precise timing perception. Differences in VOT or the duration of formant transitions that distinguish consonants are on the order of tens of milliseconds. During auditory-motor entrainment participants adjust their movement based on changes in timing of as little as 3 ms (Repp, 2000; Madison and Merker, 2004). Thus, expert entrainment performance relies upon greater precision of timing perception than is necessary for speech perception and, by extension, development of phonological awareness. As a result, entrainment practice could enhance the precision of timing perception to a greater degree than would everyday language experience, leading to benefits for language processing as well.

## Emotion

### Entraining to music is a positive emotional experience, thereby facilitating learning

Entrainment is a natural social behavior; in fact, toddlers are only able to entrain to a metronome if it is produced by another person, as opposed to a machine or a disembodied sound (Kirschner and Tomasello, 2009). Moreover, not only is entrainment facilitated when performed in a social setting, the act of entrainment strengthens social bonds. For example, a group of 4-year-old children who moved in synchrony with one another were shown to be more likely, compared to a control group, to cooperate in tasks that could be performed in either a separate or a cooperative manner (Kirschner and Tomasello, 2010). Similarly, young adults who move in synchrony with one another are led to cooperate more in subsequent economic games, compared to participants engaging in a control task involving group behavior that is not synchronized (Wiltermuth and Heath, 2009). Thus, entraining together solidifies social bonds, a process that is likely to be highly rewarding. Moreover, participants listening to music commonly make spontaneous movements even when listening by themselves, and will do so more when listening to highly rhythmic music (Janata et al., 2012), suggesting that the act of entraining to music is intrinsically rewarding even in the absence of social context. Given that emotional engagement facilitates auditory neural plasticity (David et al., 2012), the rewarding nature of entrainment during music making makes it a particularly powerful learning tool.

## Repetition

### Entrainment is a task which is constantly repeated during music practice

The process of entrainment is one of the most highly repetitive aspects of music. First, entrainment involves constantly monitoring the relationship between one’s movements and the timing of the sounds to which one is moving. In this sense, entrainment is a skill that is constantly trained throughout group practice and performance. Second, musical rhythms are highly predictable: durational patterns are repeated and tempi are maintained for long periods of time, allowing the performer and audience to predict—and move along to—sound events. As a result, even complex forms of entrainment (for example, a group producing a multilayered temporal pattern as part of a piece of music) are quite repetitive. During this repeated entrainment practice musicians are constantly monitoring the timing of acoustic events. Over time, this repeated, effortful practice hones automatic mechanisms which track auditory timing and relay this information to the motor system.

## Attention

### Successful entrainment requires focusing attention on acoustic timing

Focusing attention enhances the brain’s ability to adapt to input (Fritz et al., 2005). As a result, a musical feature upon which attention is focused during musical performance is more likely to lead to cross-domain enhancements than features which are not attended. Although a seemingly simple task, entraining to a metronome calls upon attentional resources. Children with attention deficit and hyperactivity disorder, for example, are more variable in tapping to a metronome (Pitcher et al., 2002; Rubia et al., 2003; Toplak and Tannock, 2005; Ben-Pazi et al., 2006), suggesting that an inability to sustain attention hinders mechanisms that would normally reduce tapping variability. This relationship between sustained attention ability and variability during entrainment has also been demonstrated in a typically-developing population (Tierney and Kraus, 2013b), confirming that entrainment relies upon attentional resources.

Error corrections to supraliminal perturbations during entrainment are more accurate and faster than corrections in response to subliminal perturbations, which tend to be overly large, leading to overshoot and the necessity for further corrections (Repp, 2001, 2002, 2011). Thus, optimal entrainment performance requires the focusing of attention on the timing of acoustic events. Repeated focused attention to a particular feature may enhance the automatic precision of neural encoding of that feature within the auditory midbrain in part via efferent corticofugal connections (Kraus and Chandrasekaran, 2010). Over time, repeated attention to auditory timing in the past may shape the auditory system such that representation of timing becomes automatically more precise (Kraus and Nicol, 2014).

Given that entraining to a signal as simple as an isochronous pulse relies on attention, it is likely that entrainment during performance of real music is much more demanding of attentional resources, given the much more complex rhythmic structure of music. The perception of musical structure has been hypothesized to rely upon entrainment of ongoing neural oscillations (Large, 2008), a process which has been also suggested as a mechanism of selective attention (Calderone et al., 2014). Rhythm perception could, therefore, involve a structured waxing and waning of attention as important time points (“strong” and “weak” beats) come and go (Large and Jones, 1999). This dynamic attending may enable listeners to predict when sounds are more or less likely to occur, facilitating behavioral entrainment (Patel et al., 2005). Dynamic allocation of attentional resources is, therefore, a vital component of entrainment to music (Tierney and Kraus, 2014).

## Predictions of PATH

One prediction of PATH is that musical training should lead to an enhanced ability to use durational cues during speech perception to discriminate speech sounds and segment words and phrases. Consistent with this prediction, professional musicians are better able to distinguish consonants based on durational cues such as VOT (Elmer et al., 2013; Zuk et al., 2013) and formant transition durations (Zuk et al., 2013) but are no better than nonmusicians at distinguishing consonants based on frequency information alone (Zuk et al., 2013). Musicians are also better able to detect anomalous lengthening of the final syllables of sentences (Marie et al., 2011), suggesting that they are better able to take advantage of durational cues to word and phrase segmentation. These enhancements in the perception of speech timing may be due to the entrainment practice that is commonly a part of musical training (e.g., playing with others, with recordings, or with a metronome). Given that these studies were not specifically designed to study the effects of entrainment in isolation, however, we do not know to what extent entrainment practice played a part in the training of these musicians.

A second, related prediction of PATH is that musicians whose training places greater emphasis on entrainment will show enhanced phonological skills relative to musicians whose training does not emphasize entrainment. One way to test this prediction would be to compare drummers vs. vocalists in terms of their auditory system timing and phonological abilities. The vocalists are likely to spend less time entraining with a metronome, a recording, or groups of musicians than the drummers. Intuitively, one might expect singers to have better phonological abilities than drummers (since their musical form involves words), yet PATH makes the opposite, counterintuitive prediction.

## Practical applications

It may be possible, using the auditory brainstem response to complex sounds, to identify children with imprecise neural timing who would likely benefit from musical training that emphasizes precise auditory-motor entrainment. The complex auditory brainstem response demonstrates high test-retest reliability (Hornickel et al., 2012) and tracks with individual differences in speech perception and language skills (Kraus and Chandrasekaran, 2010), making it an ideal candidate for identifying children with auditory-based communication problems. The complex auditory brainstem response can serve as a useful metric of the impact of musical training on the auditory system (Kraus and Chandrasekaran, 2010; Barrett et al., 2013; Strait and Kraus, 2014). Moreover, entrainment is among the easiest of musical skills to isolate and assess in an automated fashion. As a result, it may be practical to boost entrainment ability with video games and training programs, an approach which could benefit children unable to afford traditional music lessons.

## Other rhythmic skills

Performance is correlated across various rhythm tasks such as entrainment, rhythm discrimination, and amplitude rise time perception, (Thomson et al., 2006; Thomson and Goswami, 2008; Huss et al., 2011), suggesting that these tasks broadly assess a single rhythmic competence. Nevertheless, there is evidence that dramatic impairments in single rhythmic skills can coexist with normal performance in other rhythmic skills. Phillips-Silver et al. (2011), for example, reported a case study of a participant who was able to entrain to a metronome but not to a piece of music. Similarly, Launay et al. (2014) describe a group of participants with difficulties tapping to the beat of rhythmic patterns but with preserved ability to entrain to a metronome. On the other hand, Fries and Swihart (1990) report that a patient with right hemisphere damage was unable to entrain to an acoustic rhythm but was able to entrain to a visual stimulus and could both discriminate rhythmic patterns and produce rhythms from memory. Auditory-motor entrainment, therefore, appears to be dissociable from other rhythmic skills.

These findings suggest that there are several identifiable rhythmic skills that at least partially rely on distinct neural resources. As a result, each rhythmic skill may relate to language skills in different ways. Tracking the rhythmic structure of music, for example, may rely on phase-locking of ongoing slow oscillations (1.5–7 Hz) in auditory cortex to the rhythmic structure of music (Large, 2008); this same neural mechanism has also been proposed for the tracking of the amplitude envelope of speech (Goswami, 2011). Alternately, tracking the rhythmic structure of music may call upon motor planning regions, which could work in concert with the auditory system to predict when future beats will arrive (Patel and Iversen, 2014). This procedure could also underlie the tracking of rhythmic regularities in speech, such as the tendency for speakers to slow as they near the ends of phrases or sentences. Thus, although both entrainment and rhythmic discrimination (Strait et al., 2011) relate to language skills, the mechanisms underlying these two relationships may be different. Here we propose that auditory-motor entrainment and phonological skills relate due to a shared reliance on precise representation of neural timing in the auditory system.

## Summary

In summary, we hypothesize that musical training incorporating entrainment practice requires musicians to perceive the timing of acoustic events with a high degree of precision. This constant attention to the timing of sounds eventually leads to increased timing precision in the auditory system’s automatic representation of sound, which in turn leads to enhanced perception of the timing of speech sounds. The perception of speech timing is vital for the acquisition of phonological skills, which facilitates reading development. PATH, therefore, explains the consistent finding that musical training can lead to enhancements in reading ability as a consequence of the central role of entrainment in musical practice and performance.

## Conflict of interest statement

The authors declare that the research was conducted in the absence of any commercial or financial relationships that could be construed as a potential conflict of interest.
